# Emerging and Novel Treatments for Pituitary Tumors

**DOI:** 10.3390/jcm8081107

**Published:** 2019-07-25

**Authors:** Mirela Diana Ilie, Hélène Lasolle, Gérald Raverot

**Affiliations:** 1 INSERM U1052, CNRS UMR5286, Cancer Research Center of Lyon, 28 Laennec Street, 69008 Lyon, France; 2 “Claude Bernard” Lyon 1 University, University of Lyon, 43 “11 Novembre 1918” Boulevard, 69100 Villeurbanne, France; 3 Endocrinology Department, “C.I.Parhon” National Institute of Endocrinology, 34-36 Aviatorilor Boulevard, 011863 Bucharest, Romania; 4 “Groupement Hospitalier Est” Hospices Civils de Lyon, Endocrinology Department, Reference Center for Rare Pituitary Diseases HYPO, 59 Pinel Boulevard, 69677 Bron, France

**Keywords:** pituitary neuroendocrine tumors (PitNETs), pituitary adenoma, aggressive pituitary tumors, treatment, temozolomide, peptide receptor radionuclide therapy, bevacizumab, tyrosine kinase inhibitors, everolimus, immunotherapy

## Abstract

A subset of pituitary neuroendocrine tumors (PitNETs) have an aggressive behavior, showing resistance to treatment and/or multiple recurrences in spite of the optimal use of standard therapies (surgery, conventional medical treatments, and radiotherapy). To date, for aggressive PitNETs, temozolomide (TMZ) has been the most used therapeutic option, and has resulted in an improvement in the five-year survival rate in responders. However, given the fact that roughly only one third of patients showed a partial or complete radiological response on the first course of TMZ, and even fewer patients responded to a second course of TMZ, other treatment options are urgently needed. Emerging therapies consist predominantly of peptide receptor radionuclide therapy (20 cases), vascular endothelial growth factor receptor-targeted therapy (12 cases), tyrosine kinase inhibitors (10 cases), mammalian target of rapamycin (mTOR) inhibitors (six cases), and more recently, immune checkpoint inhibitors (one case). Here, we present the available clinical cases published in the literature for each of these treatments. The therapies that currently show the most promise (based on the achievement of partial radiological response in a certain number of cases) are immune checkpoint inhibitors, peptide receptor radionuclide therapy, and vascular endothelial growth factor receptor-targeted therapy. In the future, further improvement of these therapies and the development of other novel therapies, their use in personalized medicine, and a better understanding of combination therapies, will hopefully result in better outcomes for patients bearing aggressive PitNETs.

## 1. Introduction

Pituitary neuroendocrine tumors (PitNETs) are typically slowly progressing tumors of the anterior pituitary gland, with most cases being easily treated by medical treatment or surgery. However, some of these tumors show an aggressive behavior, being either locally invasive and rapidly growing or showing resistance and/or multiple recurrences in spite of optimal use of standard therapies (surgery, conventional medical treatments, and radiotherapy). In rare cases these tumors may even metastasize [1,2]. Therefore, alternative treatment options are needed for the treatment of aggressive PitNETs, including carcinomas. To date, temozolomide (TMZ), an oral alkylating agent, has been the most used in the clinic [1,3]. Although the use of TMZ in aggressive PitNETs, including carcinomas, has led to a remarkable improvement in the five-year survival rate in responders (both overall and progression-free) [4,5,6], and established itself as the first-line chemotherapy once standard therapies have failed [1], a significant percentage of patients either do not respond to TMZ (30% of the patients showed progressive disease (PD) on first-line TMZ in the largest published series [3]), or progress once TMZ is stopped [3]. Moreover, the response to a second course of TMZ was less effective [3], therefore other treatment options are clearly needed. Several new therapies, notably peptide receptor radionuclide therapy (PRRT), vascular endothelial growth factor receptor (VEGF)-targeted therapy, tyrosine kinase (TK) inhibitors, PI3K/Akt/mTOR pathway inhibitors, and recently, immune checkpoint inhibitors, are starting to emerge for the treatment of aggressive PitNETs. In this review, to report the response to these different treatments, and in order to standardize the available published data, we have used, where this information was available, the same criteria used by McCormack 2018 et al. in the recent European Society for Endocrinology (ESE) survey on the treatment for aggressive PitNETs: (1) For the radiological response: Complete response (CR) *=* no visible tumor, partial response (PR) = >30% tumor regression, stable disease (SD) = <30% tumor regression and <10% increase in tumor size, and progressive disease (PD) = >10% increase in tumor size or new metastases, and (2) for the biochemical response: CR = hormone level normalization, PR = >20% hormone level reduction, SD = <20% change in the hormone level, and PD = >20% hormone level increase [3].

## 2. Temozolomide (TMZ)

TMZ works by methylating the DNA, which further leads to irreversible damage of DNA and cell death. O6-methylguanine-DNA methyltransferase (MGMT), an endogenous DNA repair enzyme that removes added methyl groups, potentially counteracts TMZ cytotoxic effects [1,3,6], and therefore low levels of MGMT expression may predict a better response to TMZ treatment [6]. In glioblastomas, methylation of the *MGMT* promotor leading to gene silencing and lower levels of MGMT expression is a frequent event, and the methylation status of *MGMT* was shown to correlate with the protein expression level (determined by immunohistochemistry) and to be a reliable prognostic marker of response to TMZ [1,6,7]. On the contrary, in PitNETs, the methylation status of *MGMT* gene has not been demonstrated to correlate with the protein expression level (suggesting that other factors may impact *MGMT* gene transcription), and therefore, immunohistochemistry is currently recommended for the assessment of the MGMT status in PitNETs [1,7].

In the largest published series on TMZ, the ESE survey [3], 157 patients with aggressive PitNETs and carcinomas were treated with TMZ as first-line chemotherapy. 37% of the patients showed a radiological response (31% PR, and 6% CR), an additional 33% of the patients showed SD, and finally 30% showed PD. The maximal radiological response occurred within three months in 23% of cases and within six months in 59% of cases. In terms of the biochemical response, 19% showed CR, 34% PR, 27% SD, and 21% PD.

A better response to TMZ was recorded in patients bearing clinically functioning tumors independently of their MGMT status, these tumors being 3.3 times more likely to regress on TMZ than their non-functioning counterparts. Concerning patients’ MGMT status, patients with a low MGMT status (defined as <10%) showed a better response than patients with an intermediate MGMT status (10–50%), and both these categories responded better than those patients having a high MGMT status (>50%). Moreover, CR was recorded only in patients having a low MGMT status. Patients who received concomitant radiotherapy (either the Stupp protocol, i.e., administering six weeks of fractionated radiotherapy concomitantly with TMZ 75 mg/m^2^ daily, followed by TMZ 150–200 mg/m^2^ daily for five days every 28 days for another 6–12 months, or variations of this protocol), also showed a two-fold increase in response rate [3].

A low MGMT status has been shown not only to predict a better response to first-line TMZ [3], but also to predict survival of patients treated with first-line TMZ [7]. Moreover, in the same study [7], five patients having recurrent/progressive disease were given a second course of TMZ, of these one patient with low MGMT status showed PR, another patient showed SD, while both of the patients with high MGMT status (defined as >10%) had PD, leading to the conclusion that some patients having low MGMT status may also respond to administration of a second course of TMZ [7]. This is particularly interesting given the fact that following first-line TMZ, PD is not rare (in the ESE survey, 25% of the patients with CR, 40% of the patients with PR, and 48% of the patients with SD showed PD [3]) and that, when given a second course of TMZ, only two patients out of 18 had PR (though one received TMZ + bevacizumab), five patients out of 18 had SD (though additional treatment was given in three of them), and the remaining 11 patients had PD [3].

The ESE guidelines on aggressive PitNETs, including carcinomas [1], recommend TMZ either as monotherapy (150–200 mg/m^2^ daily for five days every 28 days) or as the Stupp protocol if patients have not previously reached maximal radiotherapy doses [1]. This treatment should be evaluated after three cycles in order to identify responders, and in responders to first-line TMZ, the treatment should then be continued for six months or more when a continued therapeutic effect is noted [1]. Data on long-term treatment with TMZ are too scarce to recommend its maintenance over a long duration however, clinical observations suggest that TMZ should be maintained as long as it is efficient in controlling tumor progression and hormonal secretion, and it is well tolerated.

The most common side-effects of TMZ in patients with PitNETs were fatigue, cytopenias, and nausea/vomiting, but other side-effects including hearing loss, headache, hypotension, edema, adrenal crisis, and abnormal liver function tests have also been reported [1,3]. The current guidelines recommend prophylactic anti-emetic therapy during five out of 28 days when TMZ is administered, a hematological profile on the 22nd day of the 28-day cycle, and monitoring of hepatic function before each TMZ cycle, halfway through the first cycle, and 2–4 weeks following cessation of the treatment. Moreover, prophylactic antibiotic therapy (pentamidine or trimethoprim-sulfamethoxazole) has been recommended in case of significant lymphopenia or in case of dose-dense regimens, concurrent radiotherapy, or hypercortisolism (due to Cushing’s syndrome or oral corticosteroids) [1].

## 3. Tyrosine Kinase (TK) Inhibitors

TK inhibitors are orally administered drugs that lead to reduced TK phosphorylation of targeted proteins [8]. In PitNETs, the use of TK inhibitors is now emerging, with the main aim of targeting the ErbB family signaling pathway. Of the four members of ErbB receptors: ErbB1 (also known as epidermal growth factor receptor (EGFR) or HER1), ErbB2 (or HER2/Neu), ErbB3 (or HER3), and ErbB4 (or HER4), the first two have been the most studied in PitNETs [9].

The existing preclinical and clinical evidence concerns mainly lactotroph and corticotroph tumors [9,10,11,12,13,14,15] and was recently reviewed by Ben-Shlomo and Cooper [9]. Both in vivo and in vitro experiments showed a decrease in cell proliferation/tumor size and/or a decrease in hormonal secretion of lactotroph and corticotroph tumors with TK inhibitor treatment [9].

Considering published cases (Table 1) [3,10,16], of eight cases treated with lapatinib, a dual ErbB1/ErbB2 inhibitor, six of these cases consisted of dopamine-resistant lactotroph tumors not previously treated with TMZ. At six months, these cases showed either SD (four cases) or PD (two cases) in terms of tumor volume, and either PR (three cases) or PD (three cases) in terms of hormonal secretion [10,16]. Four other cases of unknown tumor type, that received either lapatinib, erlotinib (an ErbB1 inhibitor [17]), or sunitib (a multitargeted TK inhibitor [18]) as a second or third line therapy after TMZ, showed PD [3].

TK inhibitor side-effects are mainly related to their simultaneous targeting of normal tissues [19]. The toxicity profile of TK inhibitors varies as a function of their target TKs [18] and of their potency [19]. Side-effects encountered with TK inhibitors include hematological events (anemia, neutropenia, and thrombocytopenia), hypertension, thrombosis/embolism, myocardial infarction, bleeding, skin rash, edema, nausea and vomiting, diarrhea, constipation, muscle cramps, bone alterations, fatigue, fever, neuropathy, and elevation of liver enzymes. Lapatinib is, in general, well-tolerated, its most common adverse effects include diarrhea (that may be dose limiting), moderate fatigue, nausea, and acneiform rash [18].

Although TK inhibitors thus far have been proposed in PitNETs for targeting of the ErbB pathway, TK inhibitors do not only target this pathway. In fact, there are more than 20 currently approved TK inhibitors, some of them with multiple targets, that can be categorized, based on their main target, into inhibitors of EGFR, VEGF receptor (VEGFR), breakpoint cluster region-Abelson kinase (Bcr-Abl), and anaplastic lymphoma kinase (ALK) [8]. Given the fact that VEGF has also been shown to be implicated in the pathogenesis of PitNETs [20], and that ALK7 was associated with an increased proliferation in lactotroph tumors [21], a more personalized choice of TK inhibitor may hopefully translate into a better response to this therapy in the future.

## 4. VEGF-Targeted Therapy

VEGF-targeted therapy is another emerging therapeutic option. Bevacizumab, an anti-VEGF monoclonal antibody, is the main option used in PitNETs, but some TK inhibitors such as sunitinib, also target the VEGFR pathway.

In a series of 148 PitNETs, six of which were carcinomas [22], Lloyd et al. found an increased VEGF expression, by immunohistochemistry, in non-treated somatotroph tumors, silent corticotroph tumors, non-oncocytic null cell tumors, and in carcinomas in comparison to the other subtypes, while VEGF expression was decreased in tumor tissue in comparison to the adjacent non-tumor tissues when the latter was present [22]. In another series of 197 PitNETs [23], 58.9% showed high VEGF expression by immunohistochemistry. This high VEGF expression was more frequently found in gonadotroph (29 cases high versus eight cases low), lactotroph (17 cases high versus 11 cases low), and in non-functioning PitNETs (40 cases high versus 30 cases low), while in corticotroph, somatotroph, and thyrotroph PitNETs, nearly half of the tumors showed high VEGF expression levels and half showed low VEGF expression [23].

To date, bevacizumab has been administered either as monotherapy [24,25] or in different combinations (with TMZ [26], with TMZ and radiotherapy [27] or with pasireotide [28]) in five cases of corticotroph tumors, of which four were carcinomas (Table 2). Four out of the five cases showed SD [24,25,26,28], while in the case where bevacizumab was associated with TMZ and radiotherapy, CR was obtained [27]. In the ESE survey [3], another seven cases treated with bevacizumab as monotherapy or in combination with TMZ were reported (Table 2), with PR, SD, or PD as the outcome [1,3], but unfortunately there were no details provided regarding the tumor type. However, it is noteworthy that from 18 cases in the ESE survey that presented with PD after the cessation of TMZ and were then administered a second course of TMZ, only two patients showed subsequent PR, of whom one received bevacizumab in combination with TMZ [3].

The side-effects of bevacizumab most commonly include hypertension and proteinuria, however arterial thrombosis, including acute myocardial infarction, venous thromboembolism, hemorrhage, gastrointestinal perforation, and wound healing complications have also been reported [29,30].

Interestingly, dopamine is also an inhibitor of VEGF signaling and has been proposed as a novel anti-angiogenic therapy that acts through the stabilization of abnormal blood vessels and in doing so also improves the efficacy of other drugs [31]. In a recent study [31], Chauvet et al. treated a mouse model of hemorrhagic lactotroph tumor with either bromocriptine alone, axitinib (a VEGFR inhibitor) alone, or with a combination of the two drugs, and found that bromocriptine not only blocked tumor growth, but also induced regression of the abnormal blood supply and normalization of the blood vessel structure. Axitinib also restrained the tumor growth and improved vascular remodeling, while only the combination of bromocriptine and axitinib therapy suppressed intratumoral hemorrhage and was able to restore blood vessel perfusion [31]. This is in line with older experiments that showed increased VEGF expression in models of dopamine-resistant lactotroph tumors: When anti-VEGF treatments were administered, both a decrease in vascularity and a decrease in proliferation/tumor size were observed [32]. Moreover, somatostatin and the somatostatin analog pasireotide were also shown to decrease the VEGF secretion of human non-functioning PitNETs in culture by 15% or more, in 13 out of 25 primary cultures studied [33].

## 5. mTOR Inhibitors

Upregulation and/or overactivation of the PI3K/Akt/mTOR pathway have been reported in human PitNETs [34,35,36] and inhibitors of this pathway were shown to have anti-tumoral effects in in vitro human PitNET cultures and in in vitro and in vivo murine models and cell lines [37,38,39,40,41].

Of the PI3K/Akt/mTOR pathway inhibitors, everolimus (EVE), an orally active mTOR inhibitor, is the only one that has been tested in patients, with six cases reported so far (Table 3) [3,42,43,44]. EVE, by binding to a protein named FKBP12, inhibits mTOR and its downstream signaling cascade, resulting in decreased protein synthesis, reduced cell growth, and cell cycle arrest [37,38]. In in vitro models of PitNETs, EVE administration resulted in the inhibition of cell proliferation, reduction of cell viability, and promotion of apoptosis [38,45].

Regarding the cases treated with EVE, in the only case not previously treated with TMZ, the patient achieved stability of tumor volume for 12 months and a decrease in prolactin levels (no data is available subsequent to this point) [44]. However, from the five cases where EVE was tried as a second- or third-line therapy in patients previously treated with TMZ, it was found to be unsuccessful in four cases [3,42], while in one case of a corticotroph carcinoma harboring an mTOR pathway mutation (STK11/F298L), the patient showed transient SD before then showing progression [43].

Preclinical studies in primary cultures from human nonfunctioning PitNETs have shown that, when associated with a somatostatin analogue (SSA)—either octreotide or pasireotide—EVE showed increased efficacy, including a change from being non-effective to effective [45,46]; the same was true in the AtT-20 (mouse corticotroph tumor) cell line with the addition of octreotide [46]. However, from the five cases treated unsuccessfully with EVE, one patient with a corticotroph carcinoma did not receive concomitant SSA treatment [43], another patient with a corticotroph carcinoma did not tolerate the SSA and therefore received only one injection [42], and for the remaining three cases [3] there is no information available on the tumor type, nor on whether EVE treatment was associated with an SSA.

In terms of side effects of EVE, the most common are usually mild and include fatigue, rash, stomatitis, diarrhea, myelosuppression, hyperglycemia, and hyperlipidemia [47,48]. However, mTOR inhibitors can also have less common side effects, such as symptomatic non-infectious pneumonitis, which can be life threatening [47].

Preclinical studies in either primary cultures from human PitNETs [40,41], or in murine models/cell lines [39,40,41] have also shown that the use of PI3K inhibitors, alone or in combination with mTOR inhibitors, hold promise for the treatment of aggressive PitNETs, and it is notable that clinically approved PI3K inhibitors already exist. Furthermore, a dual PI3K/mTOR inhibitor has been tested, either alone or in combination with TMZ, in in vivo and in vitro murine models and showed a synergistic anti-tumoral effect [49].

## 6. Immune Checkpoint Inhibitors

The recent demonstration of PitNETs (especially functioning ones) showing the presence of tumor-infiltrating T lymphocytes [50,51,52] and expressing programmed death ligand 1 (PD-L1), a potential biomarker of response to immune checkpoint inhibitors therapy [50,51], raised the hope of possible efficacy of immune checkpoint inhibitors in these tumors. Immune checkpoint inhibitors work by blocking inhibitory signals of T cell function/activation, signals that would otherwise allow tumors to evade the immune response. More precisely, these molecules work by blocking CTLA-4 or PD-1 found on T cells, or its ligand, PD-L1, found on tumor cells and antigen-presenting cells. By doing so, the immune checkpoint inhibitors enhance the function of T cells and reactivate anti-tumoral immune responses, thus favoring the destruction of tumor cells [51,53,54].

A single case [55] of a functioning corticotroph carcinoma, previously treated with TMZ and carboplatin, and bearing hypermutations suggestive of being alkylator-induced, has been reported in the literature. The therapeutic sequence prior to the use of the immune checkpoint inhibitors included two neurosurgeries, radiotherapy, two additional neurosurgeries, pasireotide, cabergoline, four cycles of TMZ in combination with capecitabine, bilateral adrenalectomy, two additional cycles of TMZ in combination with capecitabine, two cycles of etoposide and carboplatin, and again radiotherapy for the primary tumor, the immunotherapy being started shortly thereafter. The patient was treated with a combination of two immune checkpoint inhibitors, five cycles of nivolumab (an anti-PD-1 monoclonal antibody, 1 mg/kg every three weeks) in combination with ipilimumab (an anti-CTLA-4 monoclonal antibody, 3 mg/kg every three weeks). Following the five cycles with the two immune checkpoint inhibitors, the primary tumor volume decreased by 59% and the main liver metastasis volume decreased by 92%, while the levels of plasma ACTH fell from 45,550 to 66 pg/mL. After the five cycles, the patient was kept on maintenance nivolumab, and at the six months follow-up, continued to respond. The reported side effects consisted of a 40 °C fever, mild elevation of transaminases, and supposed hypophysitis (the patient already had hypopituitarism) [55].

The side effects of immune checkpoint inhibitors are immune-related adverse events. They can affect every organ, but generally the skin, the gastrointestinal tract, the liver, and the endocrine system are most affected, the patients potentially developing a rash, pruritus, vitiligo, diarrhea, colitis, hepatitis, hypophysitis, dysthyroidism, primary adrenal insufficiency, and diabetes [53,54]. Concerning the organ systems less frequently affected, side effects include sarcoidosis, inflammatory pneumonia, episcleritis, uveitis, renal insuffıciency, aseptic meningitis, transverse myelitis, Guillain–Barre syndrome, red cell aplasia, thrombocytopenia, neutropenia, and acquired hemophilia A [53].

Despite these potential side effects, immune checkpoint inhibitors represent a promising treatment option in aggressive PitNETs, especially if the patients have been previously treated with TMZ, which for the moment is the recommended first-line chemotherapy. Indeed, as we observed for the other therapeutic options, the administration of a first course of TMZ seems to not only render the tumor less responsive to a second course of TMZ, but additionally to most of the other available treatment options. In contrast, TMZ (and other conventional chemotherapies) may induce somatic hypermutations, which in turn will potentially render the tumor more responsive to immunotherapy [55]. Moreover, there are also studies that have shown that the efficacy of PD-1 inhibitors could be enhanced by concurrent radiotherapy [56]. Therefore, combining PD-1 inhibitor therapy with radiotherapy may also prove to be a therapeutic option in PitNETs in the future.

## 7. Peptide Receptor Radionuclide Therapy (PRRT)

PRRT consists of the use of radiolabeled somatostatin receptor (SSTR) binding molecules in order to target tumors expressing these receptors [57]. The rationale behind the administration of PRRT in PitNETs is the widespread expression of SSTR by different PitNET subtypes (most frequently SSTR2A and SSTR5) [58] and the demonstrated uptake of radiolabeled SSTR analogs on positron emission tomography/computed tomography (PET/CT) [1,59].

To date, the experience with PRRT in aggressive PitNETs, including pituitary carcinomas, is also limited, with only 20 cases being reported in the literature (Table 4) [3,4,60,61,62,63,64,65,66,67]. In terms of tumoral response, from these 20 cases, only three cases demonstrated PR [3,64,67] and three patients had SD [3,61,62], with one having overall SD, but showing CR in some of the nodules [62]. It is interesting to note, however, that patients showed PR or SD in all but one case not previously treated with TMZ [60], in sharp contrast to the patients previously treated with TMZ, in which the disease either progressed, or information on the outcome was lacking.

In terms of side effects, PRRT is mainly toxic for the bone marrow and kidneys. Toxicity for the bone marrow manifests usually as transient anemia, thrombocytopenia, and/or leucopenia, which must be monitored. Concerning the kidney toxicity, solutions of amino acids should be administered to confer nephroprotection [64,68].

Although PRRT seems a promising treatment option, patients with tumors expressing marginal levels of SSTR2 or where this expression is lost with the progression of the tumor, cannot at the moment benefit from this therapy [69]. In order to improve the SSTR2 targeting, Taelman et al. [69] screened for drugs able to upregulate SSTR2 and identified nine epigenetic modifiers capable of upregulating SSTR2 in vivo and in vitro, and having synergistic effects. Their method therefore holds promise for converting ineligible patients to eligible candidates [69]. Moreover, all of the currently approved PRRT represent radiolabeled SSTR analogs. However, in recent years, interest in radiolabeled SSTR antagonists has grown and increasing evidence (consisting of preclinical studies, but also human data) points to the potential superiority of radiolabeled SSTR antagonists [70]. JR11, an antagonist of SSTR showing selectivity for SSTR2, is now under clinical development, both as an imaging and a therapeutic agent [70].

## 8. Conclusions

In conclusion, in aggressive PitNETs, including carcinomas, after the failure of conventional treatments, TMZ is currently the recommended first-line treatment. Where the patients have not already reached maximal radiotherapy doses, the Stupp protocol, in which TMZ is first associated with fractionated radiotherapy, should be used [1]. Given the fact that roughly one third of patients showed a radiological response to first-line TMZ, one third showed SD, one third showed PD, and even fewer patients responded to a second course of TMZ [3], other treatment options are urgently needed. Therapies currently showing the most promise (based on achieving radiological PR in a certain number of cases) are immune checkpoint inhibitors, PRRT, and VEGF-targeted therapy, while TK inhibitors and everolimus have so far shown, at best, radiological SD. However, too few patients have been treated, and, moreover, the protocols used and therapeutic sequences varied greatly between patients, making it difficult to draw any definitive conclusion. Therefore, there is an absolute need for clinical trials for aggressive PitNETs, including carcinomas. Given the fact that these tumors are rare, it would also be important to have available “basket” studies. In the case of immune checkpoint inhibitors, there is at the moment one such basket clinical trial available on combination treatment with nivolumab and ipilimumab (NCT02834013), but only for pituitary carcinomas. Last, but not least, it is also of crucial importance to validate and standardize predictive markers of response, especially given that there appears to be a trend for a better response to most of the alternative therapies in TMZ-naïve patients, though no definitive conclusion can yet be drawn. In the future, the further development and/or improvement of these novel therapies, their personalized use, and a better understanding of combination therapies, including their correct sequencing and timing, will hopefully result in better outcomes for patients bearing aggressive PitNETs.

## Figures and Tables

**Table 1:** Cases of aggressive pituitary neuroendocrine tumors and pituitary carcinomas treated with tyrosine kinase (TK) inhibitors.

Ref.	Sex and Age at Diagnosis	Tumor Type	Carcinoma	Previous Treatments	Treatment	Response	Adverse Effects
[3]	NA	NA	NA	TMZ *	Lapatinib *	PD *	NA
[3]	NA	NA	NA	TMZ *	Lapatinib *	PD *	NA
[3]	NA	NA	NA	TMZ *	Erlotinib *	PD *	NA
[3]	NA	NA	NA	TMZ *	Sunitib *	PD *	NA
[10,16]	Female, 42y	Functioning lactotroph	No	BCT, NS, CAB 7 mg weekly	Lapatinib 1250 mg daily + CAB 7 mg weekly for 6 months	TV: SD at 6 months HS: PR at 1 month and 6 months	Alopecia (mild), diarrhea (single self-limited episode), appetite loss (mild), and rash
[10,16]	Female, 31y	Functioning lactotroph	No	CAB, BCT, NS, BCT, CAB 7 mg weekly	Lapatinib 1250 mg daily + CAB 7 mg weekly for 6 months	TV: SD at 6 months HS: PR at 1 month and 6 months	Alopecia (mild), diarrhea (few self-limiting episodes), and rash
[16]	2 females and 2 males, 19-70y	Functioning lactotroph	1 carcinoma *	NS (1 in 1 patient and 2 in 3 patients), RT in 1 patient, DA in all	Lapatinib 1250 mg daily + DA for 6 months (3 patients) and for 3 months (1 patient)	TV: SD (2 patients at 6 months), and PD (1 patient at 3 months and 1 patient at 6 months) ** HS: PR (1 patient at 6 months) and PD (1 patient at 3 months and 2 patients at 6 months)	Reversible elevation of transaminases (grade 1), rash (grade 2), and asymptomatic bradycardia (grade 1)

Reference (Ref.), not available (NA), temozolomide (TMZ), progressive disease (PD), years (y), bromocriptine (BCT), neurosurgery (NS), cabergoline (CAB), tumor volume (TV), stable disease (SD), hormonal secretion (HS), partial response (PR), RT (radiotherapy), DA (dopamine agonist); * no further information available ** as assessed by the authors (the percentage by which the tumor increased or decreased not reported)

**Table 2:** Cases of aggressive pituitary neuroendocrine tumors and pituitary carcinomas treated with VEGF-targeted therapy.

Ref.	Sex and Age at Diagnosis	Tumor Type	Carcinoma	Previous Treatments	Treatment	Response	Adverse Effects
[25]	Male, 38y	Silent corticotroph	Yes	4 NS, RT, NS, TMZ, NS, TMZ, surgery for metastases, TMZ, RT for metastases, NS	BVZ 10 mg/kg every 2 weeks for 26 months (ongoing)	TV: SD for 26 months **	NA
[28]	Female, 25y	Functioning corticotroph	Yes	2 NS, bilateral adrenalectomy, NS, RT, SSA, RT, TMZ	BVZ + pasireotide for 6 months	TV: SD at 6 months ** HS: PR at 6 months	NA
[24]	Female, 50y	Functioning corticotroph	No	2 NS, RT, NS, bilateral adrenalectomy, 2 NS, LAR+CAB, TMZ	BVZ *	Transient SD ** (patient deceased due to complications of another NS)	NA
[27]	Male, 63y	Functioning corticotroph	Yes	NS	BVZ 10 mg/kg every 2 weeks + TMZ 75 mg/m^2^ daily + RT for 2 months, then TMZ 200 mg/m^2^ daily for 5 consecutive days out of 28 days for 12 cycles	TV: CR of the pulmonary nodule 8 weeks after starting BVZ+TMZ+RT, with no recurrence for 5 years HS: NA	NA
[3]	NA	NA	NA	No previous TMZ *	BVZ + first-line TMZ *	PR *	NA
[3]	NA	NA	NA	TMZ *	BVZ + second course of TMZ *	PR *	NA
[3]	NA	NA	NA	TMZ *	BVZ + second course of TMZ *	PD *	NA
[3]	NA	NA	NA	TMZ *	BVZ + second course of TMZ *	NA	NA
[1,3]	NA	NA	NA	TMZ *	BVZ as second-line therapy *	PR after 3 months *	NA
[1,3]	NA	NA	NA	TMZ *	BVZ as second-line therapy *	SD *	NA
[1,3]	NA	NA	NA	TMZ *	BVZ as third-line therapy *	PD *	NA
[26]	Male, 50y	Functioning corticotroph adenoma transformed into silent corticotroph carcinoma	Yes	NS, RT, surgery for metastases, RT for metastases, TMZ	BVZ 10–15 mg/kg every 2 weeks (09/2010–11/2012) + TMZ 150–200 mg/m^2^ daily for 5 consecutive days monthly (09/2010–08/2011)	TV: SD for 8 years **	NA

Vascular endothelial growth factor (VEGF), reference (Ref.), years (y), neurosurgery (NS), radiotherapy (RT), temozolomide (TMZ), bevacizumab (BVZ), tumor volume (TV), stable disease (SD), not available (NA), somatostatin analogue (SSA), hormonal secretion (HS), partial response (PR), lanreotide (LAR), cabergoline (CAB), complete response (CR), progressive disease (PD); * No further information available, ** as assessed by the authors (the percentage by which the tumor increased or decreased not reported and/or hormone levels not reported).

**Table 3:** Cases of aggressive pituitary neuroendocrine tumors and pituitary carcinomas treated with everolimus (EVE).

Ref.	Sex and Age at Diagnosis	Tumor Type	Carcinoma	Previous Treatments	Treatment	Response	Adverse Effects
[42]	Male, 45y	Functioning corticotroph	Yes	2 NS, RT, bilateral adrenalectomy, RT on metastases, TMZ	EVE 5 mg daily + octreotide 30 mg for 1 month, then EVE 5 mg daily for another 2 months	No effect at 3 months **	NA
[43]	Female, 46y	Functioning corticotroph	Yes	2 NS, bilateral adrenalectomy, NS, RT, OCT, NS, OCT, NS, capecitabine + TMZ, NS	EVE 7.5 mg daily + palliative RT on metastases (01–02/2015), EVE 10 mg daily (02–06/2015), palliative RT on metastases (06–07/2015), EVE 7.5 mg daily + capecitabine 1000 mg/m^2^ b.i.d. two weeks out of three (07–09/2015)	TV: ~5 months transient stability, then PD ** HS: NA	Multifocal herpes zoster (left arm and left eye, requiring enucleation) despite prophylaxis, and neutropenia
[3]	NA	NA	NA	TMZ *	EVE as second- or third- line therapy *	PD *	NA
[3]	NA	NA	NA	TMZ *	EVE as second- or third- line therapy *	PD *	NA
[3]	NA	NA	NA	TMZ *	EVE as second- or third- line therapy *	PD *	NA
[44]	Male, 62y	Functioning lactotroph	No	NS, RT, CAB, NS, CAB 0.75 mg daily	EVE 10 mg daily + CAB 1.5 mg daily	TV: SD at 5 and 12 months HS: PR at ~20 weeks (stable at ~35 weeks)	Altered mental status due to hyperglycemia, transient hypogeusia, and mouth sores

Reference (Ref.), years (y), neurosurgery (NS), radiotherapy (RT), temozolomide (TMZ), not available (NA), octreotide (OCT), twice daily (b.i.d.), tumor volume (TV), progressive disease (PD), hormonal secretion (HS), cabergoline (CAB), stable disease (SD), partial response (PR), * no further information available, ** as assessed by the authors (the percentage by which the tumor increased or decreased not reported and/or hormone levels not reported).

**Table 4:** Cases of aggressive pituitary neuroendocrine tumors and pituitary carcinomas treated with peptide receptor radionuclide therapy (PRRT).

Ref.	Sex and Age at Diagnosis	Tumor Type	Carcinoma	Previous Treatments	Treatment, Number of Cycles (cumulative dose/activity)	Response	Adverse Effects
[60]	Female, 16y	Functioning corticotroph	Yes	2 NS, RT, 4 NS, RT, NS, bilateral adrenalectomy, NS, RT	90Y-DOTATOC, 2 cycles (200mCi)	NA, but the patient died of elevated intracranial pressure shortly after	NA
[61]	Male, 56y	NIR	No	NS, RT	177Lu-DOTATOC, 3 cycles (600 mCi)	TV: SD **	NA
[62]	Male, 40y	NF *	Yes	NS, RT, NS, surgery for metastasis	177Lu-DOTATATE, 4 cycles (~30 MBq)	TV: CR in some nodules, and overall SD at 40 months **	Transient thrombocytopenia
[62]	Male, 39y	Functioning somato-lactotroph	No	NS, RT, LAN, CAB, 4 NS with gliadel wafers in 2, TMZ, RT	177Lu-DOTATATE, 2 cycles (15.3 Mbq)	TV: PD ** (the patient died 2 months after PRRT was started—deterioration of brainstem disease) HS: NA	NA
[62]	Male, 26y	Silent corticotroph	No	2 NS, RT, NS, TMZ, TMZ, NS	177Lu-DOTATATE, 1 cycle	TV: PD **	Severe increase in the facial pain
[63]	Male, 59y	NF *	No	3 NS, RT, SSA, TMZ	177Lu-DOTATATE *	TV: NA, but the patient died in the following months	NA
[63]	Male, 46y	Functioning somatotroph	Yes	6 NS, RT, SSA/PEG, TMZ, palliative RT	90Y-DOTATATE *	No effect **	NA
[64]	Male, 23y	Functioning somatotroph	No	SSA, NS, SSA, RT	90Y-DOTATATE, 4 cycles (400mCi)	TV: PR at 12 months HS: PR at 12 months	Transient anemia and leucopenia
[4]	NA	NA	NA	TMZ *	DOTATOC *	No effect **	NA
[4]	NA	NA	NA	TMZ *	DOTATOC *	Ongoing *	NA
[3]	NA	NA	No	No previous TMZ *	As first-line therapy *	PR *	NA
[3]	NA	NA	No	No previous TMZ *	As first-line therapy *	SD *	NA
[3]	NA	NA	NA	TMZ *	As second- or third-line therapy *	PD *	NA
[3]	NA	NA	NA	TMZ *	As second- or third-line therapy *	PD *	NA
[3]	NA	NA	NA	TMZ *	As second- or third-line therapy *	PD *	NA
[3]	NA	NA	NA	TMZ *	As second- or third-line therapy *	PD *	NA
[3]	NA	NA	NA	TMZ *	As second- or third-line therapy *	PD *	NA
[65,66,67]	Female, 58y	Functioning lactotroph	No	BCT, NS, CAB, RT	111In-octreotide, 5 cycles (37 GBq) + CAB 0.5 mg daily	TV: PR after the first 2 cycles, further PR after the next 2 cycles, and further PR after the last cycle HS: PR	None
[65,66]	Male, 54y	Functioning lactotroph	No	CAB, 3 NS, RT, TMZ	177Lu-DOTATOC, 2 cycles (12.6 GBq)	TV: PD HS: Good biochemical control **	None
[65,66]	Female, 53y	NF *	No	5 NS, RT, TMZ	177Lu-DOTATOC, 5 cycles (29.8 GBq)	TV: PD	None

Reference (Ref.), years (y), neurosurgery (NS), radiotherapy (RT), 90Yttrium-DOTATOC (90Y-DOTATOC), not available (NA), non-immunoreactive (NIR), 177Lutetium-DOTATOC (177Lu-DOTATOC), tumor volume (TV), stable disease (SD), non-functioning (NF), 177Lutetium-DOTATATE (177Lu-DOTATATE), complete response (CR), lanreotide (LAR), cabergoline (CAB), bromocriptine (BCT), temozolomide (TMZ), progressive disease (PD), somatostatin analogues (SSA), pegvisomant (PEG), 90Yttrium-DOTATATE (90Y-DOTATATE), 111Indium-DTPA-octreotide (111In-octreotide), hormonal secretion (HS), partial response (PR); * no further information available, ** as assessed by the authors (the percentage by which the tumor increased or decreased not reported and/or hormone levels not reported).
